# Addressing the risk factors for the prevention of cardiovascular diseases in the Philippines

**DOI:** 10.1093/inthealth/ihae049

**Published:** 2024-07-23

**Authors:** Dalmacito A Cordero

**Affiliations:** Department of Theology and Religious Education, De La Salle University, Manila, Philippines

To the Editor,

Ischaemic heart disease topped the causes of death in the Philippines from January to July 2023. More than 65 000 cases of this ailment were recorded during those 6 months, representing 19% of all recorded deaths during that time.^1^ The disease develops when the heart's arteries fail to deliver enough oxygen-rich blood to the heart due to a buildup of plaque in the lining of the arteries.^2^ The World Health Organization (WHO) explains that most cardiovascular diseases (CVDs) can be prevented by addressing behavioural risk factors such as tobacco use, unhealthy diet and obesity, physical inactivity and harmful use of alcohol. A risk factor is a characteristic, condition or behaviour that increases the likelihood of getting a disease or injury. Risk factors are often presented individually, however, in practice they do not occur alone, often coexisting and interacting with one another.^3^ Table 1 presents the statistical data based on various surveys on the most common risk factors for CVDs in the Philippines.

**Table 1:** Risk factors and survey highlights on CVDs in the Philippines

Behavioural risk factors	Survey highlights
Tobacco use (smoking)	Currently use tobacco: 19.5% overall (15.1 million adults), 34.7% of men and 4.2% of women. Currently smoke tobacco: 18.5% overall (14.4 million adults), 33.3% of men and 3.7% of women. Currently smoke tobacco daily: 14.5% overall (11.2 million adults), 26.3% of men and 2.6% of women. Currently smoke manufactured cigarettes: 17.4% overall (13.5 million adults), 31.5% of men and 3.2% ofwomen.^8^
Unhealthy diet and obesity	27 million Filipinos are overweight or obese. For the past 2 decades, overweight and obesity among adults has almost doubled, from 20.2% in 1998 to 36.6% in 2019.^9^ Overall physical inactivity prevalence of 93.4% ranked second in the world—among 146 countries—with the most physically inactive adolescents behind South Korea.^10^
Alcohol consumption	4 in 10 Filipino adults (40.1%) reported past 30-day alcohol use. Men used more alcohol than women (51.5% vs 28.9%). One in three Filipinos (33.1%) reported high-risk and heavy alcohol use by consuming six or more alcoholic beverages in one sitting. Men engaged in more significant numbers in binge alcohol consumption than women (43.2% vs 22.9%).^11^

With the WHO emphasizing the need to address these risk factors, the Philippine government implemented new intervention programs. For smoking, the government enacted Republic Act No. 11900 (the Vaporized Nicotine and Non-Nicotine Products Regulation Act), to regulate vaporized nicotine and non-nicotine products (VNNPs) and novel tobacco products (NTPs). It also aims to reduce the harm caused by smoking and establishes a comprehensive and differentiated regulatory framework for the importation, manufacture, sale, packaging, distribution, use and communication of vaporized nicotine and non-nicotine products, along with other novel tobacco products. The law safeguards minors by restricting the sale, including the online trade, distribution and marketing of VNNPs and NTPs. It prohibits tobacco product–related activities within 100 m of schools, playgrounds and facilities frequented by minors.^4^ For alcohol consumption, the government has imposed an increased excise tax through Republic Act No. 11467 for different alcohol products.^5^ The high taxes for companies will be cascaded to consumers, causing hesitancy to buy alcohol products and thus decreasing consumption. Lastly, for unhealthy eating and obesity, multisectoral and multilevel actions are being undertaken. The National Policy on Addressing Overweight and Obesity is being developed to guide all stakeholders in curbing obesity using population-based approaches for prevention, regulatory mechanisms to influence the food environment, management of existing cases and research and surveillance. The Philippine Nutrient Profile Model will guide determining which foods and beverages can be marketed to children and as the basis for front-of-pack labelling of food products. This tool is intended to influence food manufacturers to produce healthier food for consumers.^6^

Despite these initiatives, the government needs help, cooperation and collaboration from different institutions to implement its policies and programs. Other institutions such as schools, the private sector and religious groups must help one another in the massive information dissemination about the nature, causes and risk factors of the disease. They can utilize different social media platforms to make it happen since most Filipinos are deeply immersed in social media. Families can also implement reward–incentive strategies to keep each member physically fit through group exercise activities, sports competitions and games. Caravans, campaign drives and regular celebrations that promote healthy diets must be held regularly for public awareness and free medical assistance. Non-pharmacological smoking cessation programs must be encouraged, such as individual face-to-face intervention (behavioural counselling or motivational interviewing), group intervention (group counselling or health talk), e-Health intervention (m-Health or internet-based), self-help, printed intervention and brief advice (not adopting any counselling strategy).^7^ Lastly, the government should have more infrastructure projects such as nature parks and sports facilities to promote play, exercise and other outdoor activities for everyone. These places can be avenues to enhance one's physical and mental habits and be more active, thus avoiding physical inactivity and possible obesity. With all these interventions, we can expect more positive results and have a better chance of lowering the number of cases of CVD in the Philippines.

## Data Availability

All the necessary data are available in the manuscript.
